# Dr. Jane C. Wright (1919-2013): The Architect of Modern Chemotherapy and Clinical Oncology

**DOI:** 10.7759/cureus.88511

**Published:** 2025-07-22

**Authors:** Muhammad Hamza Shah

**Affiliations:** 1 Department of Internal Medicine, Antrim Area Hospital, Antrim, GBR; 2 School of Medicine, Queen's University Belfast, Belfast, GBR; 3 Centre for Anatomy, The University of Edinburgh, Edinburgh, GBR

**Keywords:** cancer research, chemotherapy, historical vignette, medical pioneers, oncology

## Abstract

Dr. Jane Cooke Wright (1919-2013) was a trailblazing African American surgeon-scientist who transformed cancer chemotherapy from an experimental last resort into a cornerstone of modern oncology. Over a career spanning 40 years, she and her collaborators pioneered human tissue‐culture methods to test anti‐cancer drugs, introduced nitrogen mustard and methotrexate therapies, and advocated for novel multi‐drug combination regimens. Dr. Wright held major research and clinical leadership positions and was instrumental in the founding of many eminent organizations/bodies, including the Chemotherapy Department at New York Medical College and the American Society of Clinical Oncology (ASCO), which she co-founded in 1964. As an African American woman, she overcame pervasive racial and gender barriers to become the highest‐ranking Black woman physician in U.S. medicine by the 1960s. This article reviews Dr. Wright’s scientific contributions in chemotherapy development and clinical research, her personal and educational background (including the influence of her father, Dr. Louis T. Wright), and her enduring impact on cancer care and medical leadership.

## Introduction and background

**Figure 1:**
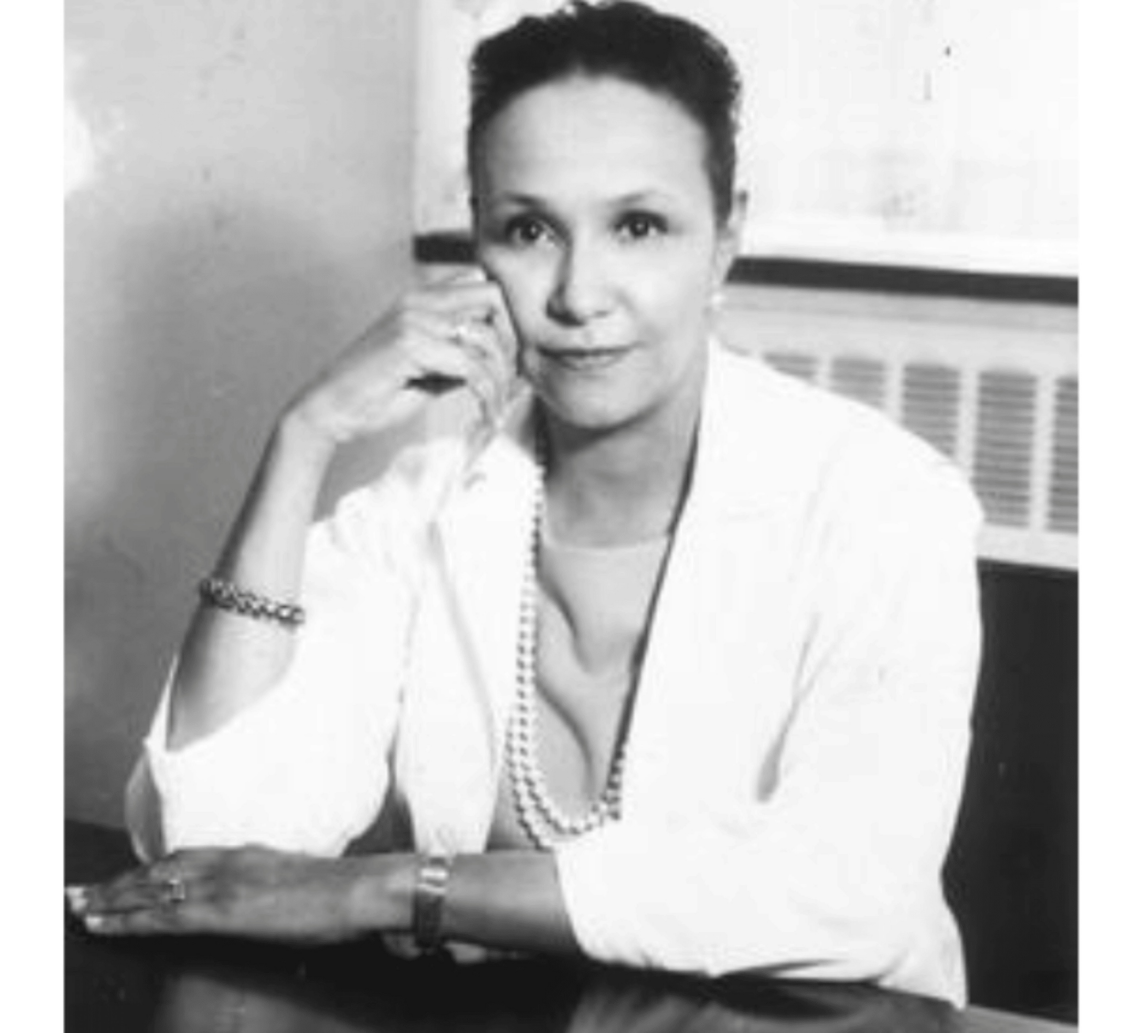
Photograph of Dr. Jane Cooke Wright Image courtesy of Wikimedia Commons (public domain): https://commons.wikimedia.org/wiki/File:Jane_Cooke_Wright.jpg

Chemotherapy was once considered a desperate measure among cancer patients - toxic, unpredictable, and largely ineffective. In the 1940s and 1950s, cancer treatment was dominated by surgery and radiation, with little hope for patients whose disease had spread [1]. Physicians and researchers grappled with toxic compounds with unpredictable effects, scant clinical data, and devastating side effects. The scientific community had yet to fully embrace the idea that cancer could be systematically studied and treated using pharmacologic tools. It was in this milieu that Dr. Jane Cooke Wright (Figure 1) helped transform the direction of oncology [2]. She brought structure and scientific method to the field of chemotherapy at a time when few believed in its potential. Dr. Wright was among the first physicians to test anticancer agents directly on human tissues rather than relying solely on animal models, leading to important discoveries such as the effectiveness of methotrexate on solid tumors [3]. She also pioneered innovative techniques for delivering chemotherapy directly to tumor sites, thereby improving drug efficacy and patient outcomes.

In addition to her scientific contributions, Dr. Wright helped shape the institutional and political landscape of modern oncology. She was the only woman and only African American among the seven founding members of the American Society of Clinical Oncology (ASCO), later serving as its first Secretary/Treasurer [4]. She advised U.S. presidents on national cancer policy, served on the National Cancer Advisory Board, and mentored a generation of oncologists, many of whom would go on to lead major institutions. Through these roles, she pushed for a more inclusive and scientifically rigorous approach to cancer care. Today, chemotherapy is seen as a core pillar of cancer treatment, often used in conjunction with surgery, radiation, immunotherapy, and targeted agents. This evolution owes much to Dr. Wright’s vision and persistence. Her legacy is not only scientific but institutional, political, and deeply personal. She redefined what was possible in cancer treatment and who could lead that charge. This article explores the scientific, institutional, and historical significance of Dr. Jane Cooke Wright’s work and her enduring impact on the field of oncology.

## Review

Personal and educational journey

Jane Cooke Wright’s scientific career was rooted in a remarkable family and educational background. Her paternal grandfather, Ceah Ketcham Wright, had been born into slavery but became a physician, instilling in the family a legacy of perseverance [5]. Her father, Louis Wright, was a civil‐rights activist as well as a pioneer physician: he was among the first Black graduates of Harvard Medical School (1915), the first African American physician appointed at NYC’s municipal Harlem Hospital, and in 1929 became the city’s first Black police surgeon [6]. Her mother, Corinne Cooke Wright, was a New York City school teacher. This family's emphasis on education and public service deeply influenced Jane Wright’s values and aspirations. Jane Wright attended Ethical Culture High School (NYC), graduating in 1938 [3]. Although her father initially encouraged her to take up medicine, she briefly pursued her interest in art - earning a Bachelor of Arts in Art from Smith College in 1942.

After college, she followed her father’s guidance, enrolling at New York Medical College. She graduated first in her class with honors in 1945, an extraordinary achievement at a time when there were very few Black physicians of either sex. Wright then completed an internal medicine internship at Bellevue Hospital (1945-46) and a surgical residency at Harlem Hospital (1947-48), temporarily taking a break from her academic and professional pursuits in 1948 for the birth of her first child [3]. In January 1949, she briefly worked as a physician for the NYC public schools but soon left to join her father at Harlem Hospital’s Cancer Research Foundation. This move reunited the two for collaborative research. In Harlem, Wright balanced laboratory work with patient care - conducting experiments by day and treating patients by night, as her father noted.

In 1950, she married David D. Jones Jr., a Harvard‐trained attorney; they eventually had two daughters, Jane and Allison (the marriage later ended in divorce) [7]. Throughout her career, Wright successfully managed family life alongside her professional duties - raising her children while traveling for research and maintaining a distinguished scientific schedule. As a Black woman in mid‐20th-century America, Wright confronted prejudice at every turn. In interviews, she recalled that her father’s own struggles (Harvard doubted he could pass the entrance exam) inspired her never to fear failure. Despite her credentials, Wright often found segregated hospitals and professional societies discouraging; yet she persisted. Notably, when she attended the ASCO founding meeting in 1964, she was the only woman and the only African American in the room [4]. In academia, she was among the first Black women appointed to professorships in surgery. By 1967, she became the highest‐ranked African American woman at any U.S. medical college. Her achievements opened doors for those who followed: Wright later became an important role model, featured on the cover of Crisis magazine [National Association for the Advancement of Colored People (NAACP) publication] in 1953, and was honored with numerous awards (including an honorary membership in Alpha Kappa Alpha Sorority and international scientific recognitions) [8].

Historical contributions to oncology and chemotherapy

From 1949 to 1952, while working at the Harlem Hospital Cancer Research Foundation under the direction of her father, Dr. Louis Wright, she helped pioneer the use of human tumor tissue cultures as a tool to guide cancer treatment decisions. By isolating malignant cells from patients and growing them in vitro, Wright was able to test the effects of various experimental drugs directly on those cells [9]. This enabled her to identify which agents were most likely to be effective for a specific individual's cancer - an early and innovative form of personalized medicine. Her approach, which matched drug regimens to tumor sensitivity in leukemia and lymphoma patients, laid critical groundwork for evidence-based chemotherapy and served as a foundation for modern therapeutic development in oncology.

In parallel with this work, Dr. Wright was also among the first clinicians to explore the use of nitrogen mustard compounds, chemical relatives of mustard gas, as a potential treatment for cancer [3,10]. Beginning in 1949, she collaborated on clinical trials that administered these agents to patients with hematological malignancies. These early efforts resulted in partial remissions, offering compelling proof that chemotherapy could deliver meaningful clinical benefits. The rationale for using mustard gas derivatives in cancer treatment stemmed from medical observations during World War I, when it was discovered that soldiers exposed to mustard gas often experienced significant depletion of white blood cells [3]. Given that leukemia is marked by uncontrolled white cell proliferation, researchers hypothesized that these compounds might be repurposed to suppress malignant growth. Dr. Wright’s clinical work helped transform this wartime observation into a viable medical treatment, bridging laboratory science and patient care. 

In 1951, her research demonstrated for the first time that methotrexate, up until then studied primarily in the context of leukemia, could also induce tumor regression in patients with advanced breast and skin cancers [11,12]. This breakthrough expanded the therapeutic scope of methotrexate and earned it a place as a cornerstone of modern chemotherapy. Building on this discovery, Dr. Wright’s laboratory went on to explore methotrexate’s effectiveness in treating a broader range of cancers, including lymphomas and sarcomas. Over the ensuing decades, methotrexate became a central component of treatment protocols for a variety of malignancies, including breast cancer, childhood leukemias, and lymphomas.

Beyond individual drug discovery, Dr. Wright was also ahead of her time in recognizing the limitations of single-agent therapies. She argued that cancer’s ability to adapt and resist treatment necessitated the use of multi-drug strategies [13]. In the 1950s and 1960s, her team began systematically testing drug combinations in rotating sequences, laying the groundwork for the concept of combination chemotherapy well before it became the standard of care. These early experiments helped establish that combining agents could dramatically improve outcomes, notably in childhood acute leukemia and Hodgkin disease. Her foresight and advocacy ultimately influenced the development of landmark treatment regimens in the 1970s, such as CMF (cyclophosphamide, methotrexate, fluorouracil) for breast cancer and MOPP (mustargen, vincristine, procarbazine, prednisone) for Hodgkin lymphoma. These regimens formed the backbone of curative therapies and remain foundational in oncology. Besides her contributions to chemotherapy strategy and drug development, Dr. Wright advanced cancer research through innovations in treatment delivery. She helped develop novel administration techniques, such as intrathecal chemotherapy for brain metastases, and led research programs at leading medical institutions - broadening the scope and impact of clinical oncology research in the United States.

After her father’s death in 1952, Wright became head of the Cancer Research Foundation at the age of 33 and continued to pursue patient-directed clinical trials. She and others also helped establish the first nationwide cancer drug trial networks when President Lyndon Johnson’s 1964 Commission on Heart Disease, Cancer, and Stroke (to which Wright was appointed) led to the creation of national cancer treatment centers [14]. In 1955, she became Associate Professor of Surgical Research at New York University and Director of Cancer Chemotherapy Research at NYU and Bellevue. In 1967, she returned to her alma mater, New York Medical College, as Professor of Surgery, head of its new Cancer Chemotherapy Department, and Associate Dean. In these roles, she established cancer research facilities, clinical cancer centers, and educational programs in chemotherapy. Wright published prolifically - over 100 peer‐reviewed papers during her career - documenting drug trials, dose schedules, and lab‐clinic correlations that went on to become part of classic oncology literature. 

Wright’s contributions also include service in leadership organizations and policy bodies. Dr. Wright’s role in co-founding the ASCO in 1964 was a major milestone in her career. At the time, medical oncology was still gaining recognition as a distinct specialty, and there were few spaces for physicians focused on cancer treatment to collaborate. Wright was invited to be one of seven founding members of ASCO, and notably, she was the only woman and the only African American in that group. Her selection reflected the respect she had earned through her clinical research and leadership. She became the organization’s first Secretary/Treasurer and played an important part in shaping its early direction [4]. What began as a small professional society eventually grew into one of the most influential cancer organizations in the world, thanks in part to the foundational work and vision of people like Wright. 

From 1966 to 1970, she served on the National Cancer Advisory Board (National Cancer Advisory Council), and she co‐chaired President Johnson’s Commission on Heart Disease, Cancer, and Stroke (1964). In 1971 (or 1977, sources differ), she became the first woman president of the New York Cancer Society. She also led international outreach - directing exchanges of oncologists with China, the Soviet Union, Africa, and leading medical missions in Ghana and Kenya in the late 1950s and early 1960s [15]. Wright’s career thus bridged laboratory science, clinical trials, education, and public health policy, solidifying the entire infrastructure of oncology treatment.

Legacy

Dr. Wright’s work has left its mark on cancer treatment and medical history. Clinically, her research findings form a part of the standard oncology canon: e.g., studies on the relationship between tumor tissue culture response and patient outcomes (a technique she refined) remain foundational in understanding drug efficacy. Her 100+ publications and pioneering clinical trials are cited in oncology textbooks as key early evidence of drug activity in human cancers. Institutionally, Wright’s leadership helped establish modern cancer care. As outlined before, her role in President Johnson’s cancer commission helped launch the National Cancer Act and the network of cancer centers. Moreover, as the first female president of the New York Cancer Society, she broadened the participation of women and minorities in cancer advocacy. Wright’s personal legacy endures in honors that memorialize her name. ASCO’s Conquer Cancer Foundation established the Jane C. Wright, M.D., Young Investigator Award in 2011, given annually to promising oncology trainees [16]. Her life is celebrated by professional societies, medical schools, and historians as emblematic of both scientific excellence and social progress. 

In 2012, Dr. Edith Mitchell of Thomas Jefferson University aptly called Wright “a true pioneer” whose achievements “changed the practice of medicine” [8]. The National Medical Association listed her among “Women Pioneers of Medical Research,” and she is often referred to as the “Mother of Chemotherapy”. Beyond these accolades, Wright’s most enduring legacy is the ongoing impact of her contributions on patient care. Many current chemotherapy regimens trace their origins to her early protocols. Clinicians and researchers continue to use tissue‐culture chemosensitivity tests, combination therapies, and principles she championed. Generations of oncologists cite her perseverance and scientific rigor as inspiration. As oncology advances into targeted and personalized therapies, Wright’s integrative approach - applying laboratory science directly to patient treatment - remains a guiding model.

## Conclusions

Dr. Wright’s career exemplified how scientific brilliance and personal courage can converge to transform a field. She started in segregated hospitals in the 1940s and rose through the ranks to the national cancer commissions by the 1960s. By demonstrating that cell‐based experiments could guide effective chemotherapy, Wright helped transform modern oncology as we know it. Her story also highlights the intersection of medicine and social justice: as an African American woman, she expanded opportunities for underrepresented groups in science and medicine. Wright passed away in 2013 at the age of 93, but her influence lives on every time a cancer patient receives a chemotherapy regimen, first pioneered by her lab, or an individual serves in a society she helped create. Her life and work embody the impact one dedicated physician-scientist can have on public health and on the course of medical history.
